# Genome report: Genome sequence of tuliptree scale, *Toumeyella liriodendri* (Gmelin), an ornamental pest insect

**DOI:** 10.1093/g3journal/jkae231

**Published:** 2024-09-27

**Authors:** Andrew J Mongue, Amanda Markee, Ethan Grebler, Tracy Liesenfelt, Erin C Powell

**Affiliations:** Department of Entomology and Nematology, University of Florida, Gainesville, FL 32611, USA; Department of Entomology and Nematology, University of Florida, Gainesville, FL 32611, USA; American Museum of Natural History, New York, NY 10024, USA; Department of Entomology and Nematology, University of Florida, Gainesville, FL 32611, USA; Department of Entomology and Nematology, University of Florida, Gainesville, FL 32611, USA; Division of Plant Industry, Florida Department of Agriculture and Consumer Services, Florida State Collection of Arthropods, Gainesville, FL 32608, USA

**Keywords:** tuliptree scale, scale insect, paternal genome elimination, Coccoidea, Coccomorpha, non-mendelian genetics, Hemiptera, chromosome-level assembly

## Abstract

Scale insects are of interest both to basic researchers for their unique reproductive biology and to applied researchers for their pest status. In spite of this interest, there remain few genomic resources for this group of insects. To begin addressing this lack of data, we present the genome sequence of tuliptree scale, *Toumeyella liriodendri* (Gmelin) (Hemiptera: Coccomorpha: Coccidae). The genome assembly spans 536 Mb, with over 96% of sequence assembled into one of 17 chromosomal scaffolds. We characterize roughly 66% of this sequence as repetitive and annotate 16,508 protein-coding genes. Then we use the reference genome to explore the phylogeny of soft scales (Coccidae) and evolution of karyotype within the family. We find that *T. liriodendri* is an early-diverging soft scale, less closely related to most sequenced soft scales than a species of the family Aclerdidae is. This molecular result corroborates a previous, morphology-based phylogenetic placement of Aclerdidae within Coccidae. In terms of genome structure, *T. liriodendri* has nearly twice as many chromosomes as the only other soft scale assembled to the chromosome level, *Ericerus pela* (Chavannes). In comparing the two, we find that chromosome number evolution can largely be explained by simple fissions rather than more complex rearrangements. These genomic natural history observations lay a foundation for further exploration of this unique group of insects.

## Introduction

Scale insects are a uniquely interesting group of arthropods, as they hold great importance for both basic and applied research. On the basic side, this group displays remarkable reproductive diversity (Gavrilov 2007; Blackmon *et al.* 2017; Ross *et al.* 2022), from familiar sexual reproduction with chromosomal sex determination (Blackmon *et al.* 2017) to hermaphroditism (Hughes-Schrader 1925; Mongue *et al.* 2021) and a complex form of purely autosomal haplodiploidy known as paternal genome elimination (PGE) (Nur 1980). Very little is known about either the molecular mechanisms of these alternative sex determination systems or how the clade transitioned between them. On the applied side, many scale insects are globally invasive pests that require intervention to control (Caltagirone and Doutt 1989; Waterhouse 1991; García Morales *et al.* 2016), but others are both geographically limited and specialized on one or a few host plants (*e.g.* Spanish moss ensign scale; Morrison 1925). Understanding the factors that differentiate these 2 groups will help better target screening and management practices. Specifically, both applied and basic research goals are held back by a lack of modern genomic resources compared with other insect clades (*e.g.* Lepidoptera; Wright *et al.* 2024). In service of beginning to address this scarcity, we have sequenced the genome of the tuliptree scale.

**Fig. 1.**
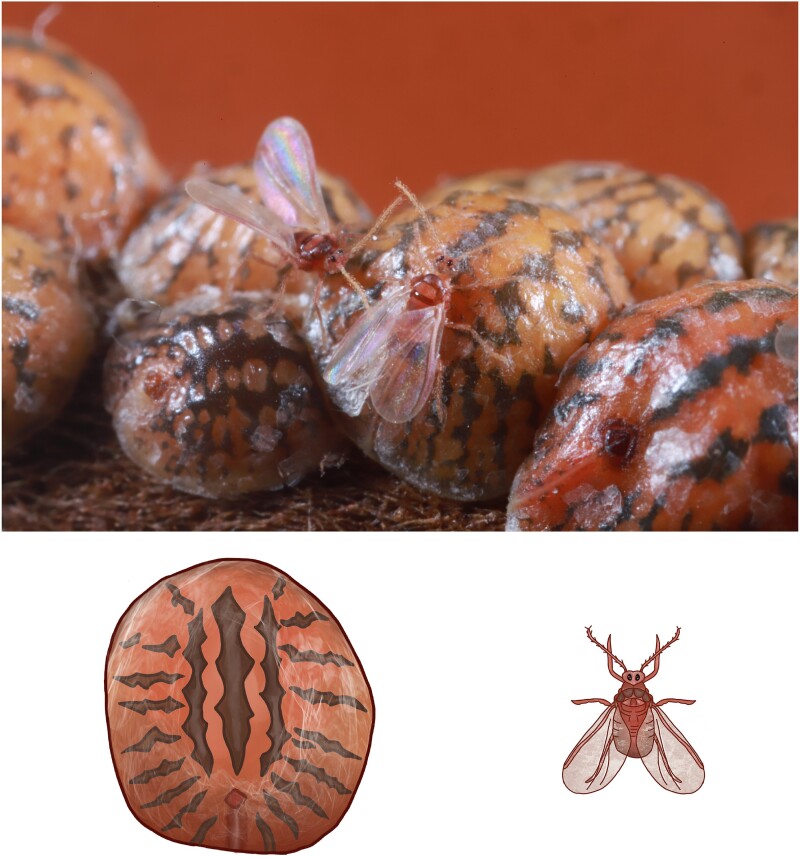
Adult *T. liriodendri* display extreme sexual dimorphism. Males (winged) are not only smaller but possess many features (wings, eyes) that are not present in adult females (larger patterned domed individuals). Top: Photograph of a colony of *T. liriodendri* with 2 males and 8 females. Bottom: Rendering of individual female (left) and male (right) from a dorsal view to highlight sexual dimorphism. Photograph by E. Powell, illustration by T. Liesenfelt.

*Toumeyella liriodendri* (Gmelin 1790), or tuliptree scale, is a soft scale insect (Hemiptera: Coccomorpha: Coccidae). It reproduces sexually, but with a fully autosomal genome and sex determined by PGE (Nur 1980). Under this system, females are fully diploid in karyotype and gene expression, but males silence and ultimately discard their paternally inherited chromosomes, making them functionally haploid (Gavrilov 2007). Unlike better-studied mealybugs (Pseudococcidae; Ross *et al.* 2010, 2024; de la Filia *et al.* 2021), which keep the entire silenced paternal genome in somatic cells, soft scales employ a variant of PGE in which some paternal chromosomes are lost during cell division, leading to a variable karyotype between cells within the same male (Gavrilov 2007). Coincident with this rare sex determination system, adults are extremely sexually dimorphic, with large, long-lived, sessile females and small, winged, non-feeding males (Fig. 1).

Ecologically, tuliptree scales are presumed native to the eastern United States and likely introduced to California (Hamon and Williams 1984) and Cuba (Novoa *et al.* 2011). This species can be a major pest of native trees including tulip tree, *Liriodendron tulipifera* L., which is commonly grown for timber, and trees in the genus *Magnolia* L., which are popular ornamental and shade trees (Burns and Donley 1970; Hamon and Williams 1984; Gill 1988). While this species prefers Magnoliaceae, it will feed on a variety of hosts across several families (Hamon and Williams 1984; Gill 1988; Miller and Williams 1995).To support the study of both the evolution of PGE and monitoring of a tree pest, we present a chromosome-level assembly of *T. liriodendri*.

## Materials and methods

### Sample collection and identification

We collected adult female *T. liriodendri* from a single colony feeding on *Magnolia grandiflora* L. (Magnoliaceae) in Gainesville, Florida, USA (29.686122, −82.345128), on 2023 June 28. We confirmed species identity with slide-mounted specimens following Hamon and Williams (1984) and Kondo and Williams (2008). Specimens were deposited in the Florida State Collection of Arthropods (FSCA) in Gainesville, Florida (barcodes FSCA_00072042–FSCA_00072046).

### DNA extraction and sequencing

We followed a sequencing strategy that has proven successful in other recent insect genome sequencing projects, including other scale insects (Mongue *et al.* 2024; Ross *et al.* 2024). First, we extracted DNA from a single adult female using a modified OmniPrep extraction protocol (G-Biosciences, St Louis, MO, USA); we followed the manufacturer's protocol for solid tissue, but extended digestion with proteinase K to overnight (∼15 h) and extended the DNA precipitation step to 1 h at −20°C. After quality checking, we sequenced genomic DNA at the University of Florida's Interdisciplinary Center for Biotechnology Research on the PacBio Sequel IIe platform (Menlo Park, CA, USA) to generate HiFi long reads from a single SMRTCell. Because of the limited yield of DNA from a single individual, we chose a low-input library preparation strategy that did not include DNA fragment size selection; this approach was a calculated tradeoff to avoid introducing additional haplotypes into the primary assembly. High molecular weight genomic DNA preparations were evaluated for read length distribution using the Agilent TapeStation, using a high-sensitivity genomic tape. DNA preparations (3–5 μg) were cleaned using the MoBio PowerClean DNA Cleanup Kit (# 12877-50). Samples were fragmented to the desired 12–15 kb range using G-tubes (Covaris Inc., # 520079). Following sample fragmentation, DNA was concentrated using AMPure beads, and ∼3 μg of clean DNA was used for Sequel IIe library construction following the manufacturer’s protocol, for a 30 h movie run. Separately, we pooled adult and juvenile females from the same colony, flash-froze them in liquid nitrogen, and sent tissue to Novogene, Inc. (Sacramento, CA, USA) to generate Arima Hi-C-linked reads (Carlsbad, CA, USA) for scaffolding. We completed the initial HiFi sequencing and genome size estimation (below) before Hi-C sequencing to ensure we chose adequate coverage depth for the latter.

### Genome size estimate

Because little is known about *T. liriodendri* and tissue samples were limited and used for genomic DNA extraction, we sought to estimate the expected genome size directly from the raw PacBio reads by counting k-mer frequencies. To do so, we used Jellyfish v2.3.0 (Marcais and Kingsford 2012) to generate frequencies and then used custom R scripts to estimate the genome size from these data.

### Genome assembly

We assembled raw HiFi reads with hifiasm v0.16.1 (Cheng *et al.* 2021), first with all parameters set to defaults and then with the “-l 3” stringency parameter to more aggressively purge duplicated haplotigs. Based on higher contiguity of the latter approach (L50 = 97 vs 95), we proceeded with the more aggressively purged assembly. At this stage we performed additional curation steps, both characterizing potential co-bionts and contaminants using blobtools v1.0 (Laetsch and Blaxter 2017) and searching for any remaining haplotigs using the purge_dups tool (https://github.com/dfguan/purge_dups), which searches for potential duplicate sequences based on coverage depth of input reads and self-alignment of the assembly; this approach did not identify any duplicate sequences. We also explored circular contigs to identify the mitochondrial sequence and potential co-bionts. We assessed baseline genome completeness with BUSCO v4.1.4 using the hemipteraodb10 dataset (Manni *et al.* 2021). With these steps complete, we aligned Hi-C-linked reads to the assembly using Arima's pipeline (found at https://github.com/ArimaGenomics/mapping_pipeline/): briefly, we independently aligned left and right reads using bwa-mem2 v2.2.1 (Vasimuddin *et al.* 2019) and filtered out chimeric reads while keeping the 5′ end using scripts from the Arima git repository and following the User Guide pdf file. We then merged separate alignment files and removed PCR and optical duplicates using Picard tools v2.25.5's MergeSamFiles and MarkDuplicates functions (“Picard toolkit” 2019). We input this curated alignment into YaHS v1.1 (Zhou *et al.* 2023). YaHS generated an initial scaffolded assembly, which we further explored by using the “juicer pre” command to generate JBAT files for visualizing HiC contacts in Juicebox 2.17 (Durand *et al.* 2016) for manual correction of the assembly. After visual inspection and correction of misplacements, we saved the updated linkage file and input it into YaHS to run “juicer post” to update the assembly to reflect changes made in Juicebox (Zhou *et al.* 2023). We again assessed BUSCO completeness. Based on the failure of the purge_dups approach to identify haplotigs in the primary assembly, we used BUSCO information to screen haplotigs as follows. We concatenated a list of all scaffolds with single-copy BUSCO sequences as well as a list of those with multicopy (duplicate) sequences. We compared the 2 lists and identified 68 non-chromosomal scaffolds that contained only duplicates and no single-copy BUSCOs. We filtered to remove these from our assembly and proceeded to repeat masking and annotation.

### Repeat masking

We characterized repeats in this final curated genome as follows. First, we modeled repeats de novo using RepeatModeler v2.0 (Flynn *et al.* 2020) including a search for long terminal repeats using the “-LTRStruct” parameter. This generated a set of species-specific repeats, which we concatenated to the end of a custom library which consisted of the 2020 Repbase arthropod and hemipteran repeat databases (Bao *et al.* 2015), combined with repeats identified with RepeatModeler in other high-quality genomes: *Icerya purchasi* Maskell (Mongue *et al.* 2024), *Planococcus citri* (Risso) (Ross *et al.* 2024), and another soft scale *Ericerus pela* (Chavannes) (Yang *et al.* 2019). We imported this curated database of hemipteran repeats augmented with scale insect specific and *T. liriodendri* specific repeats into RepeatMasker v4.0.9 (Smit *et al.* 2019) to generate a final soft-masked assembly and summary of repetitive elements.

### Gene annotation

Lacking enough samples to generate an RNAseq dataset, we chose a de novo approach to gene annotation, using the machine learning tool helixer (Holst *et al.* 2023). Helixer requires only a genome sequence and a general lineage (in our case “invertebrate”) to annotate. We passed the softmasked chromosomal assembly to helixer for annotation but note that the helixer tool claims to not be impacted by the presence or absence of masking.

### Phylogeny of Coccidae

We sought to use our newly generated genome to explore phylogenetic relationships between soft scale species. To do so, we downloaded existing datasets for other soft scales and outgroups, as shown in Table 1. To analyze the transcriptomic data, we first assembled the transcriptomes using Trinitiy v2.9.0 (Grabherr *et al.* 2011), then ran BUSCO v4.1.4 in transcriptome mode (Manni *et al.* 2021) to extract single-copy orthologs for phylogenetic inference; for genome assemblies, we ran BUSCO (Manni *et al.* 2021) directly on the genomes. Next, we used a BUSCO_phylogenomics pipeline (https://github.com/jamiemcg/BUSCO_phylogenomics) to gather single-copy BUSCOs present in at least 75% of our sample species and used FastTree v2.1.11 (Price *et al.* 2009) to create individual gene trees for each protein sequence using the JTT model of evolution (Jones *et al.* 1992) with the CAT approximation for different rates at each site (Stamatakis 2006). Then we used ASTER’s (https://github.com/chaoszhang/ASTER) ASTRAL tool (Zhang *et al.* 2018) to generate a consensus species tree with the “-R” more subsampling and placements options, and choosing *P. citri* to root as the outgroup. We assessed confidence in the tree with the local posterior probability using a quartet-based algorithm (Sayyari and Mirarab 2016).

**Table 1.** Summary of currently available soft scale sequence data as well as 2 outgroups from other families: Aclerdidae and Pseudococcidae.

Species	Family	Data type	Accession
*Aclerda* sp.	Aclerdidae	Transcriptome	SRR1821892
*Planococcus citri*	Pseudococcidae	Genome	GCA_950023065.1
*Ericerus pela*	Coccidae	Genome	GCA_011428145.1
*Ceroplastes cirripediformis*	Coccidae	Transcriptome	SRR1821905
*Coccus* sp.	Coccidae	Transcriptome	SRR1821911
*Parthenolecanium corni*	Coccidae	Genome	GCA_038050395.1

We used this dataset to explore phylogenetic relationships within the family Coccidae.

### Karyotype evolution between scale insect species

To date, scale insects of the better-studied mealybug family Pseudococcidae with chromosome-level genome assemblies all have shown a karyotype of *n* = 5 (Gavrilov 2007; Li *et al.* 2020; Ross *et al.* 2024), but with additional chromosome-level resources for scale insects comes the opportunity to explore how genome architecture has evolved. We sought to compare our newly generated *T. liriodendri* assembly (*n* = 17) with that of *E. pela* (*n* = 9; Chen *et al.* 2021). For this analysis, we removed shorter scaffolds, leaving only the chromosomal pseudomolecule scaffolds and ran Satsuma v2 (Grabherr *et al.* 2010) SatsumaSynteny2 to find orthologous matches across the genome. We then processed these matches with “BlockDisplaySatsuma” and visualized them with “ChromosomePaint” to understand the relationship between karyotypes.

## Results and discussion

### Sequencing and assembly

We generated 31 Gb of raw PacBio HiFi reads (raw data accessions found in Table 2), with a mean read length of 5,221 bp and a median quality score of 43. We first used these data to set expectations of genome size using k-mer counting analyses. Our analyses suggested we generated an average of 31 × coverage on a roughly 515 Mb genome. The karyotype of *T. liriodendri* has not been previously reported, but the contact map of the downstream assembly showed 17 clear linkage groups, even before curation, suggesting the same karyotype as *Neolecanium cornuparvum* (Thro) (Gavrilov 2007). After manual curation of the contact map, 96.5% of the total assembled length (517,894,882 bp) is contained in one of 17 chromosomal scaffolds (Table 3). The size of the genome assigned to chromosomes matches well with the k-mer-based genome size estimates, but we note that at present, we lack an estimate of genome size from flow cytometry to independently validate the genome size of *T. liriodendri*.

**Table 2.** Location of all primary and assembly data generated for *T. liriodendri*.

Data	Accession (Bioproject: PRJNA1100599)
PacBio HiFi reads	SRX24290617
Illumina Hi-C reads	SRX24290618
Assembly	GCA_041937245.1
Annotation	DOI:10.5281/zenodo.12611068

**Table 3.** Assembly statistics.

Assembly statistics	Primary HiFi assembly (Hifiasm)	Hi-C scaffolded assembly (YaHS)	Final curated and haplotig purged
Total length	543,812,424 bp	543,870,724 bp	536,198,248 bp
Sequence count	869	435	367
N50	1,574,055 bp	30,207,406 bp	30,207,406 bp
L50	95	8	8
GC content	35.44%	35.44%	35.44%
N content	0 (0.00%)	58,300 (0.01%)	58,300 (0.01%)
BUSCO completeness (hemipteraodb10, *n* = 2510)	C: 90.2% [S: 77.8%, D: 12.4%], F: 2.6%, M: 7.2%	C: 90.3% [S: 79.4%, D: 10.9%], F: 2.6%, M: 7.1%	Genome mode: C: 90.0% [S: 82.6%, D:7.4%], F: 2.7%, M: 7.3% Annotation: C: 90.4% [S: 81.8%, D: 8.6%], F: 2.1%, M: 7.5%

Assembly size, contiguity, and BUSCO completeness for the primary HiFi assembly, the HiC scaffolded assembly, and the final curated assembly.

In the context of other scale insects, there are no closely related species, *i.e.* congeners, for comparison to set expectations for genome size. That said, the closest well-characterized genome is the soft scale *E. pela*, which has a genome of 650 Mb (Yang *et al.* 2019). Other better-studied scale insects in the mealybug family Pseudococcidae have genomes of roughly 400 Mb (Vea *et al.* 2021; Ross *et al.* 2024). From this perspective, the ∼540 Mb genome of *T. liriodendri* fits well within the established trend and serves to highlight that the scale insect *I. purchasi* is an outlier with a genome of over 1 Gb in length (Mongue *et al.* 2024), likely due to its unique reproductive ecology as a self-fertile hermaphrodite (Mongue *et al.* 2021).

Working with the final curated assembly (the far right column of Table 2), we used a combination of de novo repeat finding and matching against a database of known hemipteran repeats. In total, we masked 66% of the genome, with results from RepeatMasker summarized in Table 4. This repeat percentage is higher than the ∼55% reported from the related *E. pela* (Yang *et al.* 2019), despite the smaller genome size of *T. liriodendri.* This difference is partially attributable to our use of a larger hemipteran repeat database, as applying it to the *E. pela* genome masked ∼60% of bases, but it does not fully explain the increase in repeats.

**Table 4.** Summary of masked repeats in the *T. liriodendri* genome.

Repetitive element	Count	Sequence length (bp)	Percentage of sequence
SINEs	3,162	529,083	0.10
LINEs	36,639	10,916,041	2.04
LINE1	3,326	1,083,625	0.20
LINE2	1,587	515,529	0.10
L3/CR1	1,298	423,116	0.08
LTR elements	109,745	47,806,576	8.92
ERVL	4	253	0.00
ERV Class I	15,834	4,356,406	0.81
ERB Class II	53	93,459	0.02
DNA elements	79,859	16,639,012	3.10
hAT-Charlie	75	5,663	0.00
TcMar-Tigger	13,514	1,954,000	0.36
Unclassified	1,270,206	270,951,242	50.53
Total interspersed repeats		34,684,195	64.69
Small RNA	1,961	398,467	0.07
Satellites	56	10,072	0.00
Simple repeats	86,079	4,594,864	0.86
Low complexity	11,037	586,349	0.11%

Output is based on the RepeatMasker summary table.

Finally, gene content in scale insects is also poorly characterized. On the low end, annotations of the genome of *E. pela* and *Phenacoccus solenopsis* Tinsley report 12,022 genes (Yang *et al.* 2019) and 11,880 genes (Li *et al.* 2020), respectively. On the high end, species cataloged on the web resource Mealybug Base (https://ensembl.mealybug.org/index.html) range from ∼22,000 [*Pseudococcus longispinus* (Targioni Tozzetti)] to ∼40,000 (*P. citri*) predicted genes. Our annotation of *T. liriodendri* is closer to the low end, with 16,508 protein-coding genes. At present, we lack RNA evidence to directly validate our gene predictions, but this lower number is in line with expectations from other insects. Moreover, the 8.6% BUSCO duplication we observe is very similar to the 8.0% observed in the new chromosome-level assembly of *P. citri* (Ross *et al.* 2024), which has yet to be formally annotated. Future studies of *T. liriodendri* could improve genetic resources by focusing on generating expression data and revisiting the annotation with this added line of evidence.

### Coccidae phylogeny

**Fig. 2.**
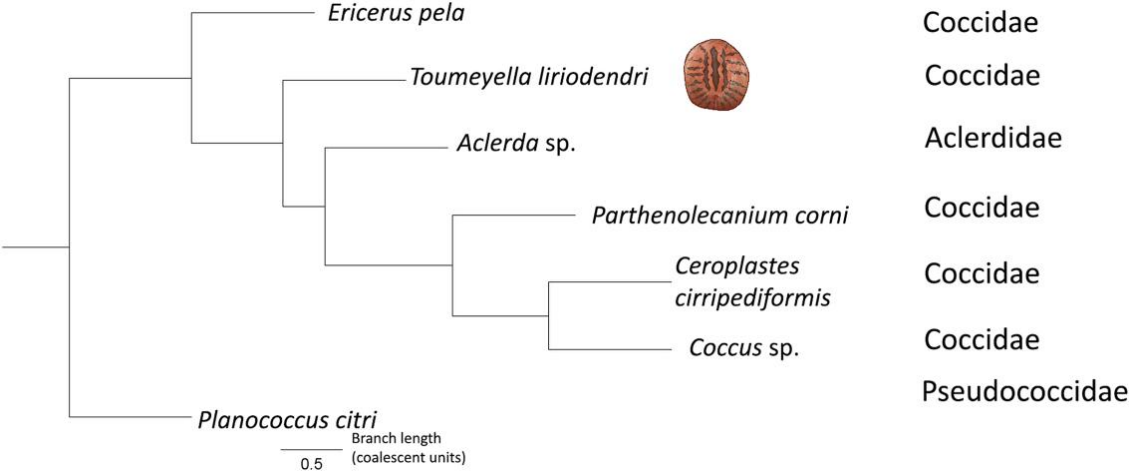
Molecular phylogeny of available Coccidae species. *T. liriodendri* is an early-diverging soft scale (indicated with a graphic), with *E. pela* as the outgroup to the rest of the family. The only sequenced *Aclerda* species is nested within the family Coccidae. All branches have a local posterior probability of 1.

We used available genomic resources to explore the phylogenetic relationship of sequenced coccid species. Specifically, we used conserved hemipteran BUSCO ortholog sequences to build 2,136 gene trees from which we inferred the overall species tree. We found that *E. pela* is the outgroup to other sequenced soft scales, with *T. liriodendri* being the next branching species (Fig. 2). Interestingly, we recover the unspecified *Aclerda* species from Johnson *et al*. (2018) within the Coccidae, despite its placement in the family Aclerdidae; a larger, mostly morphological phylogeny of scale insects also recovered Aclerdidae within Coccidae (Vea and Grimaldi 2016). This congruence of molecular and morphological results suggests that the classification of Aclerdidae and/or Coccidae may require revision.

### Soft scale karyotype evolution

**Fig. 3.**
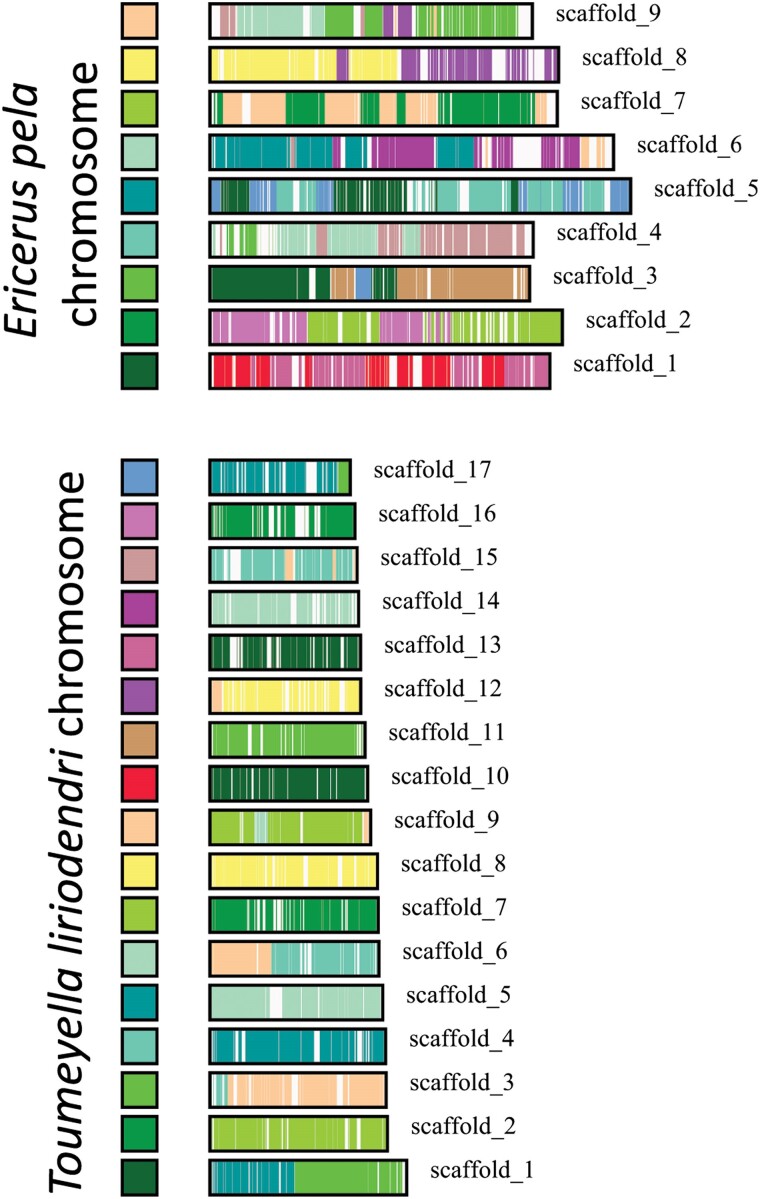
Macrosynteny between the 2 chromosome-level coccid assemblies. Investigation of synteny between the *E. pela* (*n* = 9, top) and *T. liriodendri* (*n* = 17, bottom) genomes. Colored blocks on the left of each chromosome in a given panel encode the color of a syntenic match to a chromosome in the other species [*e.g. T. liriodendri* scaffold 10 (red) matches exclusively to *E. pela* scaffold 1 (dark green)]. Overall, karyotype evolution follows a pattern of chromosomal fissions: a given *T. liriodendri* chromosome matches to only one *E. pela* chromosome, but that same *E. pela* chromosome matches to multiple *T. liriodendri* chromosomes.

Scale insect genome architecture evolution is poorly studied, but our newly generated genomic resources can begin to make sense of the differences in chromosome number between lineages. Members of Coccidae range in karyotype from *n* = 5 to *n* = 18 chromosomes, based on currently characterized species (Gavrilov 2007), with *n* = 17 for *T. liriodendri* and *n* = 9 for *E. pela* based on inference from the genome assemblies. We explored synteny, the conservation of features along chromosomes, between the 2 species and found high levels of conservation despite the different karyotypes (Fig. 3, colored blocks). It is not worth speculating on the exact series of events that created these differences, because with only 2 chromosome-level genomes for Coccidae, either the *T. liriodendri* karyotype or the *E. pela* karyotype (or indeed both) may be more recently derived compared with the genome of their most recent common ancestor. More genomic resources will be necessary to confidently resolve this history.

That said, it does not appear to be a particularly complex history based on our preliminary observations. By our count, 15 of 17 *T. liriodendri* chromosomes appear to be mainly fragments of single *E. pela* chromosomes. For instance, all of *T. liriodendri* chromosomes 8 and 12 map to 2 halves of chromosome 8 in *E. pela*, which could be explained by a simple fission event (or fusion, depending on the ancestral karyotype). The 2 exceptions, *T. liriodendri* chromosomes 1 and 6 map to 2 different *E. pela* chromosomes, indicating fusion/fission events in the opposite direction of the prevailing pattern. On top of this are more localized regions of incongruence that may represent gene trafficking or small-scale rearrangements. What drives these genomic changes between scale insect species remains an unanswered question.

This variability may be partly explained by the fact that scale insect chromosomes are holocentric, *i.e.* homologous pairs align along the length of the chromosome rather than at a single centromere point (Parida and Ghosh 1986; Gavrilov 2007). It has been argued that this chromosomal system is more permissive of fusions and fissions, as distinct karyotypes can still successfully align during cell division (Márquez-Corro *et al.* 2019); however, overall rates of chromosome number evolution do not appear to be higher in clades with holocentric taxa (Ruckman *et al.* 2020), and in another group of holocentric insects, the Lepidoptera, chromosome number appears to be remarkably conserved, with the exception of fusions involving the sex chromosome (Mongue *et al.* 2017; Wright *et al.* 2024). Thus, other factors must impact the propensity of chromosome number to vary between scale insect species. Indeed, in chromosomally sex-determined taxa, genomic rearrangements involving the sex chromosomes (*e.g.* X or Z chromosomes) may have some adaptive benefit (Mongue *et al.* 2022) but also come with the cost of potential dosage problems between males and females that requires the evolution of novel gene regulation (Gu *et al.* 2019). For a purely autosomal system such as PGE, in contrast, there is no clear adaptive advantage to chromosomal rearrangements *per se*, but the genome-wide consistency of ploidy may also make it more permissive of fusions and fissions. In either case, a broader sampling across scale insect families will be required to test these predictions.

### Conclusion

Here, we report a chromosome-level assembly for the ornamental pest tuliptree scale, *T. liriodendri*. This resource will open avenues in both basic research of reproductive diversity of scale insects as well as tracking and management of this ornamental pest. To demonstrate its usefulness, we perform preliminary phylogenetic and synteny analyses with available soft scale data. A deeper exploration of the molecular mechanisms and consequences of PGE will require more data, but this genome assembly is a key component.

## Data Availability

All data used in this project are summarized with appropriate accessions and DOIs in Table 1 (for existing data used in comparisons) and Table 2 (newly generated sequence data, genome, annotation data). Ancillary scripts can be found at https://github.com/amongue/ToliGenome/.
